# Cultivation of microalgae in food processing effluent for pollution attenuation and astaxanthin production: a review of technological innovation and downstream application

**DOI:** 10.3389/fbioe.2024.1365514

**Published:** 2024-03-20

**Authors:** Xiaowei Zhang, Qian Lu

**Affiliations:** School of Grain Science and Technology, Jiangsu University of Science and Technology, Zhenjiang, China

**Keywords:** microalgae, astaxanthin, food processing effluent, bioproduct, valorization

## Abstract

Valorization of food processing effluent (FPE) by microalgae cultivation for astaxanthin production is regarded as a potential strategy to solve the environmental pollution of food processing industry and promote the development of eco-friendly agriculture. In this review paper, microalgal species which have the potential to be employed for astaxanthin in FPE were identified. Additionally, in terms of CO_2_ emission, the performances of microalgae cultivation and traditional methods for FPE remediation were compared. Thirdly, an in-depth discussion of some innovative technologies, which may be employed to lower the total cost, improve the nutrient profile of FPE, and enhance the astaxanthin synthesis, was provided. Finally, specific effects of dietary supplementation of algal astaxanthin on the growth rate, immune response, and pigmentation of animals were discussed. Based on the discussion of this work, the cultivation of microalgae in FPE for astaxanthin production is a value-adding process which can bring environmental benefits and ecological benefits to the food processing industry and agriculture. Particularly, technological innovations in recent years are promoting the shift of this new idea from academic research to practical application. In the coming future, with the reduction of the total cost of algal astaxanthin, policy support from the governments, and further improvement of the innovative technologies, the concept of growing microalgae in FPE for astaxanthin will be more applicable in the industry.

## 1 Introduction

As a ketocarotenoid with superior antioxidative activity, astaxanthin (3,3′-dihydroxy-β, β-carotene-4,4′-dione), has been widely used in cosmetics, animal feed, and healthcare food (Wang et al., 2013; Zhao et al., 2019b; Lu et al., 2021). In recent years, some microalgal strains, such as *Chlorella zofingiensis*, *Haematococcus lacustris* (former name *Haematococcus pluvialis*), and *Chlorella sorokiniana*, have been employed for the production of natural astaxanthin (Lu et al., 2021). The research interests cover the artificial mutagenesis of algal strains, optimization of environmental factors, extraction of astaxanthin from algae biomass, antioxidative functions of algal astaxanthin and so on (Xie et al., 2018; Fernando et al., 2021; Szczepanik et al., 2022). In a real-world application, however, high cost of natural astaxanthin production by microalgae cultivation has not been fully addressed. In the market, high sale price (US $2,000–2,500 per kilogram) of natural astaxanthin is hindering the wide application of this valuable bio-product in modern agriculture (Liu et al., 2013; Lu and Lu, 2022).

The fast growths of global population and food production are accompanied with the generation of a high volume of food processing effluent (FPE) enriched with organics (Yang K. et al., 2015; Haque et al., 2017). For example, the production of 1 ton palm oil could generate 2.5–3.0 m^3^ FPE and the processing of 1 ton soybean for bean curd production can yield 7–10 tons of wastewater (Li S. et al., 2019; Chong et al., 2021). Traditionally, FPE is treated by physical filtration, aeration, anaerobic digestion, and/or chemical oxidation, yielding a large amount of sludge and greenhouse gases (CO_2_ and CH_4_) (Leifeld et al., 2018; Aziz et al., 2019; Asgharnejad et al., 2021). Compared to the traditional methods, algae-based FPE treatment, which could convert the wastewater-borne nutrients to algal biomass, has much less carbon footprint and more economic benefits. In fact, due to the excellent performance of microalgae in wastewater treatment and water recycling, microalgae cultivation has been regarded as a promising strategy to address the water pollution in agriculture, aquaculture, and industry, achieving the United Nations Sustainable Development Goal 6-“Ensure access to water and sanitation for all” (Oliveira et al., 2022; Olabi et al., 2023). Therefore, to promote the eco-friendly development of food industry, valorization of the FPE by microalgae cultivation for high-value components production is emerging into the limelight (Nishshanka G. K. S. H. et al., 2021).

To support the wide use of astaxanthin in agriculture, the safety of the harvested algal biomass is an important concern. Compared to other wastewater, such as mining effluent, chemical wastewater, and municipal wastewater, FPE contains no or much less toxic components (Lu et al., 2015; Lu et al., 2016). Accordingly, astaxanthin-rich microalgae cultivated in FPE would not be contaminated by heavy metals or chemical toxins. On one hand, in the FPE, due to the absence of toxic components, the growth of microalgae would not be seriously limited. On the other hand, biomass containing no toxic components could be widely used in agriculture. Otherwise, the toxic components may threaten human’s health through the accumulation in food chain. Microalgae have been successfully used to recover nutrients from food wastes or digested food wastes. According to previous studies, high removal efficiency of nutrient (nitrogen, phosphorus, ammonia, organic carbon, *etc.*) in different types of FPEs, such as starch processing wastewater, raw cheese whey, and household food waste, was achieved by microalgae cultivation (Schanes et al., 2018; Chuka-ogwude et al., 2020). Hence, it has been fully verified that it is a practical way to cultivate microalgae for nutrient recovery from FPE and high-value ingredient production.

In addition to the microalgae-based astaxanthin production, the application of astaxanthin-rich biomass to feed livestock, fish, shrimp, and poultry in agricultural is becoming increasingly popular. Recently, more and more beneficial effects of algal astaxanthin on animals’ health have been reported (Xie et al., 2018; Xie et al., 2020). For example, it was discovered that daily supplementation of astaxanthin-rich microalgae in diet could enhance the growth of poultry, livestock, and fish (Kumar et al., 2019; Xie et al., 2020; Awadh and Zangana, 2021). Besides, the immune response of animals can be improved by the addition of astaxanthin in diet (Xie et al., 2018). In this case, the health problems caused by the overuse of antibiotics and medicines in animal feeds can be solved. Algal astaxanthin could also enhance the pigmentation, which one of the most important quality criteria dictating the market value, of some animals, particularly poultry and fish (Vissio et al., 2021). Hence, the practical application of microalgal astaxanthin is of importance to the development of eco-friendly and value-added agriculture.

The optimization of environmental factors to induce the astaxanthin biosynthesis of algae grown in wastewater has been summarized in many previous review papers (Lu et al., 2021; Basiony et al., 2022). In terms of microalgae-based FPE treatment for astaxanthin production, there are some other interesting and important questions. Firstly, what are the microalgal species that can be employed for simultaneous nutrient recovery and astaxanthin production in FPE? Secondly, what are advantages of microalgae-based FPE treatment over traditional treatment methods in terms of profitability and carbon footprint? Thirdly, are there some technological innovations that can enhance the biomass production and lower the total cost of microalgae-based FPE treatment? Fourthly, what are the downstream applications of astaxanthin-rich microalgae in animals farming? This review paper is to provide answers to the aforementioned questions by summarizing the recent progresses and conducting in-depth analysis. It is expected that this review paper can help readers understand the promising prospects of the algae-based astaxanthin production in FPE and the downstream application of algal astaxanthin in agriculture.

## 2 Microalgal species for astaxanthin production in food processing effluent

In nature, there are a number of microalgae which could synthesize astaxanthin, but only a few algal species, such as *H. lacustris*, *Chlorococcum* sp., *C. sorokiniana*, and *C. zofingiensis*, have been intensively studied (Zhang and Lee, 2001; Chen et al., 2009; Raman and Mohamad, 2012; Wang et al., 2013). Some microalgae, such as *Oedocladium carolinianum*, *Monoraphidium* sp., *Coelastrum* sp., *Bracteacoccus aggregatus*, and *Coelastrella rubescens* are able to synthesize astaxanthin although they have not been widely commercialized (Table 1). Some of the newly isolated astaxanthin-rich microalgae are found to have high possibility of commercial application. For example, *B. aggregatus* isolated from the White Sea coastal zone (Russia) has a very high percentage (48%) of astaxanthin in the total carotenoids (Chekanov et al., 2021). In the future, with the technological development, more microalgal strains with the capacity of synthesizing astaxanthin will be found in the nature.

**TABLE 1:** Microalgal species for astaxanthin production.

Microalgal species	Source	Biomass yield (g/L)	Astaxanthin content or astaxanthin yield	References
*H. lacustris*	Obtained from the Scandinavian Culture Collection of Algae and Protozoa at the University of Copenhagen, Denmark	∼4.8 g L^-1^	2.7% of biomass (dry weight)	Wang et al. (2013)
*H. lacustris*	Obtained from Lugu Lake, Yunnan, China	∼1.38 g L^-1^	21.5 mg g^-1^	Zhao et al. (2019b)
*Chlorococcum* sp	Isolated from flora collected from the rocky wall of Taiping Mountain, Hong Kong	*NA ^a^*	23.2% of total carotenoids	Yuan et al. (2002)
*Tetraselmis* sp	Obtained from Algaetech Ltd., Malaysia	*NA*	∼5.0 mg L^-1^	Raman and Mohamad (2012)
*C. zofingiensis*	Obtained from the American Type Culture Collection, Maryland, United States	∼8–10 g L^-1^	∼8.5–10.5 mg L^-1^	Chen et al. (2009)
*Chlorococcum* sp	Selected from the preserved algae	∼20 g L^-1^	1.77 mg g^-1^ dry weight	Zhang and Lee (2001)
*C. zofingiensis*	Obtained from the Culture Collection of Algae at University of Texas, Texas, United States	0.62 g L^-1^	6.02 mg g^-1^ dry biomass	Pelah et al. (2004)
*H. lacustris*	*NA*	945 mg L^-1^	32.74 mg g^-1^	Christian et al. (2018)
*C. sorokiniana*	Obtained from Algaetech Ltd., Malaysia	*NA*	∼9.8 mg L^-1^	Raman and Mohamad (2012)
*C. zofingiensis*	Purchased from the Culture Collection of Algae at University of Texas, Texas, United States	7.5 g L^-1^	4.89 mg g^-1^ dry weight	Liu et al. (2016)
*Scenedesmus acutus*	Obtained from Iranian National Center of Genetic Resources, Iran	452 × 10 cells mL^-1^	9.4 ng cell^-1^	Khalili et al. (2019)
*Oedocladium carolinianum*	Obtained from the Freshwater Algae Culture Collection of the Institute of Hydrobiology, Chinese Academy of Sciences, Wuhan, China	4.10 g L^-1^	3.91% dwt	Wang et al. (2022)
*C. sorokiniana*	Obtained from Iranian National Center of Genetic Resources, Iran	265 × 10 cells mL^-1^	31 ng cell^-1^	Khalili et al. (2019)
*Monoraphidium sp*	Isolated from a freshwater environment at Kuala Selangor Nature Park, Malaysia	*NA*	0.476 μg mL^-1^	Kaha et al. (2021)
*Coelastrum sp*	Isolated from water samples collected from a freshwater environment at Kuala Selangor Nature Park, Malaysia	*NA*	0.999 μg mL^-1^	Kaha et al. (2021)
*Bracteacoccus aggregatus*	Isolated from the White Sea coastal zone, Russia	100–200 mg L^-1^ day^-1^	48% of total carotenoids	Chekanov et al. (2021)
*Coelastrella rubescens*	Obtained from the collection of microalgae of the Timiryazev Institute of Plant Physiology, Russian Academy of Sciences, Russia	∼0.78 g L^-1^	26.21% of total carotenoids (Astaxanthin FA monoesters: 18.26%; Astaxanthin FA diesters: 7.95%)	Minyuk et al. (2017)
*Synechocystis sp*	Genetically engineered (China)	*NA*	29.6 mg g^-1^ (dry cell weight)	Diao et al. (2020)
*Synechocystis sp*	Genetically engineered (China)	5.92 g L^-1^	4.81 mg g^-1^ (dry weight)	Liu et al. (2019)
*Chlamydomonas reinhardtii*	Genetically engineered (China)	0.62 g L^-1^	94.9 fg cell^-1^	Perozeni et al. (2020)

^a^ *NA* means not available.

In the industrial application, to select the most appropriate microalgal strain for astaxanthin production in FPE, three factors, including the source of microalgae, astaxanthin content and resistance to harsh environment, should be taken into consideration. Firstly, in the practice, microalgal strains which could be obtained from commercial organizations are more likely to be widely used. Table 1 shows that the some algal strains, including *H. lacustris*, *C. sorokiniana*, and *C. zofingiensis* have been preserved by some international commercial organizations, enabling the wide application of these microalgae for astaxanthin production in the industry. Secondly, to achieve high profitability of microalgae-based astaxanthin production, algal strains with higher content of astaxanthin are preferred. Due to the different metabolic pathways and growth conditions, astaxanthin content in microalgae varies. As shown in Table 1, astaxanthin yield in *H. lacustris* could reach 30.94 mg L^-1^ while that in *Tetraselmis* sp. was only 5.0 mg L^-1^. Low content of astaxanthin in microalgae not only results in the low productivity, but also increases the cost of astaxanthin extraction. Thirdly, algal strains with high resistance to the harsh environment are more likely to grow well in FPE and convert wastewater-borne nutrients to high-value components. In contrast, the cultivation of unrobust microalgae in FPE may result in the failure of wastewater treatment and astaxanthin production.

Based on the criteria mentioned above, in the view of the present authors, *H. lacustris*, and *C. zofingiensis* could be regarded as promising algal strains for FPE treatment and astaxanthin production. Firstly, *H. lacustris* and *C. zofingiensis* have been successfully commercialized and they can be easily purchased from some international commercial organizations (Pelah et al., 2004; Wang et al., 2013). Besides, *H. lacustris* and *C. zofingiensis*, which have been intensively studied in academic research, could be obtained from the universities or research institutes (Table 1). Since *H. lacustris* and *C. zofingiensis* could be obtained from diverse sources of microalgal strains, they have the potential of being cultivated for astaxanthin production in the industry. Secondly, *H. lacustris* and *C. zofingiensis* contain high yields of astaxanthin, reaching 30.94 and 36.68 mg L^-1^, respectively (Liu et al., 2016; Christian et al., 2018). Compared with other microalgal strains (*Tetraselmis* sp., *Chlorococcum* sp., *C. sorokiniana*, *etc.*), *H. lacustris* and *C. zofingiensis* have great advantages in the astaxanthin content and astaxanthin yield (Table 1). Thirdly, *H. lacustris* and *C. zofingiensis* could grow well in a variety of waste streams, confirming their high resistance to harsh environment in wastewater (Table 2). For example, it was reported that the tolerance level of Chlorophyceae, to which *H. lacustris* and *C. zofingiensis* belong, to ammonia toxicity is 23.758 mM, which is much higher than those of Bacillariophyceae (also known as Diatomophyceae), Dinophyceae, Prymnesiophyceae, and Raphidophyceae (Collos and Harrison, 2014). Therefore, *H. lacustris* and *C. zofingiensis* are regarded as promising microalgal strains for simultaneous FPE remediation and astaxanthin production.

**TABLE 2:** Nutrient removal by microalgae grown in food industry wastewater.

Algal species	Wastewater	Nutrient removal efficiency			Biomass yield (g L^-1^)	Astaxanthin yield	References
		COD	TN (%)	TP (%)			
*C. zofingiensis*	Palm oil mill effluent (2.5% dilution)	28.0%	64.9	86.7	0.48 g L^-1^	2.71 mg L^-1^	Fernando et al. (2021)
	Dairy industry wastewater	85.05%	93.64	98.45	1.55 g L^-1^	NA^a^	Yang et al. (2015a)
*H. lacustris*	Palm oil mill effluent (7.5% dilution)	50.9%	49.3	69.4	0.52 g L^-1^	22.43 mg L^-1^	Fernando et al. (2021)
	15% walnut shell extracts	39.18%	52.15	45.46	0.92 g L^-1^	27.17 mg L^-1^	Yu et al. (2021)
	Potato juice wastewater (Pretreated through the mesophilic methanogenic reactor)	50.1%	83.4	86.5	0.41 g L^-1^	11.5 mg g^-1^	Pan et al. (2021)
	Potato juice wastewater (Pretreated through the acidification reactor)	61.0%	71.3	92.0	0.38 g L^-1^	24.5 mg g^-1^	Pan et al. (2021)
	Corn kernels fermentation wastewater	67.0% (TOC)	91.7	100	2.028 g L^-1^	1.109 mg g^-1^ dry weight	Haque et al. (2017)
	Synthetic dairy wastewater	95.5%	79.6	56.5	0.55 g L^-1^	2.1%	Nishshanka et al. (2021a), Nishshanka et al. (2021b)
*C. sorokiniana*	Snack industry wastewater	84.3%	84.7	54.7	1.566 g L^-1^	*NA*	Gupta and Pawar (2018)
	Acid-producing wastewater	74.44%	88.05	82.69	5.67 g L^-1^	*NA*	Su et al. (2022)

^a^ *NA* means not available.

In addition to the natural microalgae strains mentioned above, some genetically engineered microalgae are able to produce astaxanthin. Through introducing genes regulating astaxanthin biosynthesis into the microalgal chassis cells, some microalgae, such as *Synechocystis* sp. and *Chlamydomonas reinhardtii*, with the capacity of synthesizing astaxanthin were obtained (Liu et al., 2019; Liang et al., 2023). As reported by the previous study, genetically engineered *Synechocystis* sp. contained high content of astaxanthin, reaching 29.6 mg g^-1^ (dry cell weight) (Lu et al., 2023b). Hence, the development of genetic modification techniques is bringing new prospects of microalgae-based astaxanthin production. However, it should be noted that due to the potential safety risks of genetic modification, genetically engineered microalgae are studied in lab research at present while have not been widely used in the industrial application.

## 3 Microalgae-based nutrient recovery in food processing effluent

### 3.1 Growth of astaxanthin-rich microalgae for FPE treatment

As the effluent of food processing industry, FPE is enriched with a variety of organics, such as carbohydrate, oil, protein hydrolysate, *etc.* As shown in Table 3, compared with the artificial mixotrophic medium, some FPEs contain much higher concentrations of total organic carbon (TOC), chemical oxygen demand (COD), total nitrogen (TN), total ammonia nitrogen (TAN), and total phosphorus (TP). Therefore, FPE may cause serious environmental pollution if it is not treated properly. With the discharge of FPE into natural waters, the enrichment of nutrients, including organic carbon, nitrogen, and phosphorus and so on, in natural waters will cause the water eutrophication (Preisner et al., 2021). As a consequence, the occurrence of algal bloom followed by eutrophication reduces dissolved oxygen content, releases toxins, and disturbs the ecological balance (Misra et al., 2011; Watson et al., 2016). Also, FPE can be regarded as a nutrient-rich substrate for microalgae cultivation. In this case, microalgae-based FPE remediation could not only attenuate the potential environmental pollution, but also yield high-value biomass, generating both environmental benefit and economic benefit.

**TABLE 3:** Biochemical properties of food processing wastewater.

Wastewater	Biochemical properties					References
	TOC (mg L^-1^)	COD (mg L^-1^)	TN (mg L^-1^)	TAN (mg L^-1^)	TP (mg L^-1^)	
Soybean fermentation effluent	2042.20	6749.33	394.44	372.44	46.78	Deng et al. (2021)
Soybean processing wastewater	*NA ^a^*	13,215	267.1	52.1	56.3	Hongyang et al. (2011)
Molasses	1870	6500	420	∼18	NA	Avcioglu et al. (2011)
Meat processing wastewater	*NA*	19,580	137.5	*NA*	10.8	Hu et al. (2019)
Meat processing wastewater	*NA*	8,470	219.8	*NA*	108.3	Hu et al. (2019)
Fruits and vegetables processing wastewater	*NA*	10,913–22300	220–252	*NA*	20.8–512.4	Asgharnejad et al. (2021)
Coffee processing wastewater	*NA*	15,040–18080	22.4–40.32	*NA*	4–7.3	Asgharnejad et al. (2021)
Meat processing wastewater	*NA*	3,560	327.6	193.0	46.8	Lu et al. (2015)
Edible oil wastewater	*NA*	15,000–100000	180–1,400	*NA*	180	Lee et al. (2019b), Asgharnejad et al. (2021)
Sugarcane spent wash	205	21,698	182	*NA*	2.15	Vasistha et al. (2023)
Dairy wastewater	*NA*	191,000	3,570	429	22,350	Lu et al. (2016)
Molasses wastewater	*NA*	4,150	864.7 (NO_3_-N: 44.7 mg L^-1^)	820	*NA*	Tsioptsias et al. (2015)
Alcohol wastewater	*NA*	65,000	618.68	279.72	47.16	Yang et al. (2015b)
TAP medium (artificial medium)	*NA*	3,870	364.4	132.0	28.6	Yang et al. (2006)

^a^ *NA* means not available.

Compared with other algal bio-products, such as bio-oil and single-cell protein, algal astaxanthin has higher values in the market. In recent years, therefore, robust algal strains, particularly *H. lacustris* and *C. zofingiensis*, have been intensively cultivated in FPE for astaxanthin production. As presented in Table 2, productivity of algal astaxanthin in some FPEs could reach over 20 mg g^-1^, confirming the feasibility of using FPE as a substrate for microalgae cultivation and astaxanthin production (Pan et al., 2021; Yu et al., 2021).

According to previous studies, high concentration of organic carbon and high ratio of C/N are favorable to the biosynthesis of astaxanthin in algal cells (Lu et al., 2021; Lu and Lu, 2022). Firstly, organic matters, such as sugars and acids, are added in artificial medium to promote the synthesis of algal astaxanthin (Ip and Chen, 2005; Chen et al., 2009). It was discovered that with the increase of glucose content in medium from 5 g L^-1^–50 g L^-1^, astaxanthin yield of *C. zofingiensis* was improved from 0.76 mg L^-1^–10.29 mg L^-1^ (Ip and Chen, 2005). It should be noted that in FPE, some organic matters are not in the forms of sugars or acids and some organic matters are even could not be directly assimilated by algal cells (Lu et al., 2015). Hence, conversion of organic matters in FPE to digestible sugars or acids is of importance to the production of algal astaxanthin. Secondly, astaxanthin formation is accompanied with the encystment induced under the high ratio of C/N (Kakizono et al., 1992; Li et al., 2022). Traditionally, to grow microalgae for astaxanthin production, low concentration of nitrate, ammonia, or organic nitrogen was added in artificial medium (Lu and Lu, 2022). As shown in Table 3, however, many FPEs, such as soybean processing effluent and meat processing effluent, contain high concentration of TN. Accordingly, in FPEs with low ratios of C/N, the synthesis of algal astaxanthin may be hindered. To achieve high productivity of microalgae biomass, nutrient profiles of some FPEs should be modified properly.

Based on the discussion above, FPEs contain essential nutrients for microalgae cultivation, but some unfavorable factors, such as the high contents of indigestible nutrients and high ratio of N/C, impact the biomass production, nutrient recovery, and astaxanthin accumulation, hindering the microalgae-based astaxanthin production in FPEs.

### 3.2 Fate of carbon in FPE treatment

In recent years, to prevent the exacerbation of climatic disasters caused by the greenhouse effect, governments are taking measures to reduce the carbon footprint of industries, including the wastewater treatment industry. Carbon neutrality has become one of the development targets of most countries in the world (Jia and Lin, 2021).

**FIGURE 1:**
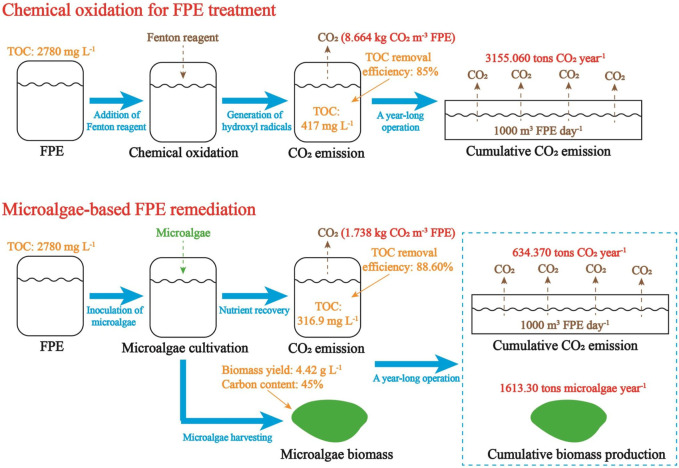
The fates of carbon in wastewater treatment based on chemical oxidation and microalgae cultivation (A wastewater treatment plant with a daily capacity of 1,000 m^3^ FPE is used as an example for the estimation of carbon emission and two different treatment technologies, traditional chemical oxidation and microalgae-based remediation, are compared).

Figure 1 demonstrates the fates of carbon in FPE treated by chemical oxidation or microalgae cultivation. In this work, to intuitively demonstrate the amount of carbon emission during the treatment of FPE, a wastewater treatment plant with a daily capacity of 1,000 m^3^ FPE is used as an example. Normally, such a wastewater treatment plant could treat the FPE produced by a large-sized food processing factory. Some data in previous studies were used to estimate the fate of carbon in FPE treatment (Lu et al., 2023b; Fajardo-Puerto et al., 2023). Concentration of organic carbon in FPE was set as 2,780 mg L^-1^. In the Fenton-based FPE treatment, the removal efficiency of organic carbon was set as 85%. In the microalgae-based FPE remediation, removal efficiency of organic carbon, biomass yield, and carbon content in biomass were set as 88.60%, 4.42 g L^-1^, and 45%, respectively.

As shown in Figure 1, in Fenton reaction, a typical chemical oxidation for organics-rich effluent treatment, with the generation of hydroxyl radicals (OH•), organic carbon contained in FPE is converted to CO_2_ (Fajardo-Puerto et al., 2023). Accordingly, 8.664 kg CO_2_, which is equal around 4.382 m^3^ CO_2_ to at 20°C, could be produced during the treatment of 1 m^3^ FPE. In a traditional wastewater treatment plant (WWTP) with a treatment capacity of 1,000 m^3^ FPE day^-1^, 3155.060 tons CO_2_ would be produced annually. Therefore, the contribution of CO_2_ emission in FPE treatment to the greenhouse effect is not neglectable.

According to the parameters mentioned above, in algae-based FPE remediation, a high portion of organic carbon was converted to biomass while CO_2_ emission was only 1.738 kg m^-3^. If this eco-friendly strategy is implemented in the WWTP to treat 1,000 m^3^ FPE daily, annual CO_2_ emission would be 634.370 tons and 1613.30 tons high-value biomass would be produced each year (Figure 1). In this case, CO_2_ emission of microalgae-based FPE remediation is 79.89% lower than that of FPE treatment by chemical oxidation. Accordingly, microalgae-based FPE remediation with much less CO_2_ emission could be regarded as a more eco-friendly and profitable strategy to solve the pollution caused by organics-rich effluent from food industry.

## 4 Technological innovations for microalgae-based FPE treatment

Microalgae-based astaxanthin production in FPE is an eco-friendly and economic strategy for waste resource recycling and high-value compound production. However, it is challenged by a couple of problems, including low removal efficiency of nutrient, high cost of biomass harvesting, and ammonia toxicity, in the practice. Firstly, due to the enrichment of undissolved organics in wastewater, in some cases, nutrient removal efficiency in microalgae-based FPE remediation is not high. For example, *H. lacustris* only removed 39.18% COD, 52.15% TN, and 45.46% TP in walnut shell extracts during the 13-day treatment (Yu et al., 2021). Under this situation, FPE after treatment still contains high concentrations of residual nutrients, which not only cause secondary pollution, but also result in underutilization of resource (Hu et al., 2021). Secondly, microalgal cells suspended in culture media or waste stream were mainly harvested by centrifugation, filtration, or Al^3+^-based flocculation (Matter et al., 2019; Najjar and Abu-Shamleh, 2020). In the practice, high energy consumption of centrifugation would dramatically increase the total cost of algal biomass and addition of Al^3+^ may limit the use of algal biomass in food and feed industry (Macías-Montes et al., 2021). Thirdly, high concentration of ammonia in some FPEs could negatively impact the growth of microalgae or even cause the failure of microalgae cultivation. To attenuate ammonia toxicity and utilize FPEs as the substrate for algae production, dilution and ammonia stripping were widely applied by previous studies (Fernando et al., 2021; Lu et al., 2023a). Nevertheless, due to the high consumption of freshwater and intensive emission of ammonia, the aforementioned two methods could not be regarded as an eco-friendly or sustainable way for microalgae-based FPE remediation.

Fortunately, in recent years, innovative technologies are developed to enhance the nutrient removal, simplify the biomass harvesting process, and alleviate the ammonia toxicity in wastewater. In this section, co-cultivation of microalgae with heterotrophic microorganisms, immobilization of microalgae by biofilm, and zeolite-based ammonia adsorption, which might promote the practical application of microalgae-based FPE valorization and astaxanthin production, were introduced.

### 4.1 Co-culture of microalgae with heterotrophic microorganisms

**FIGURE 2:**
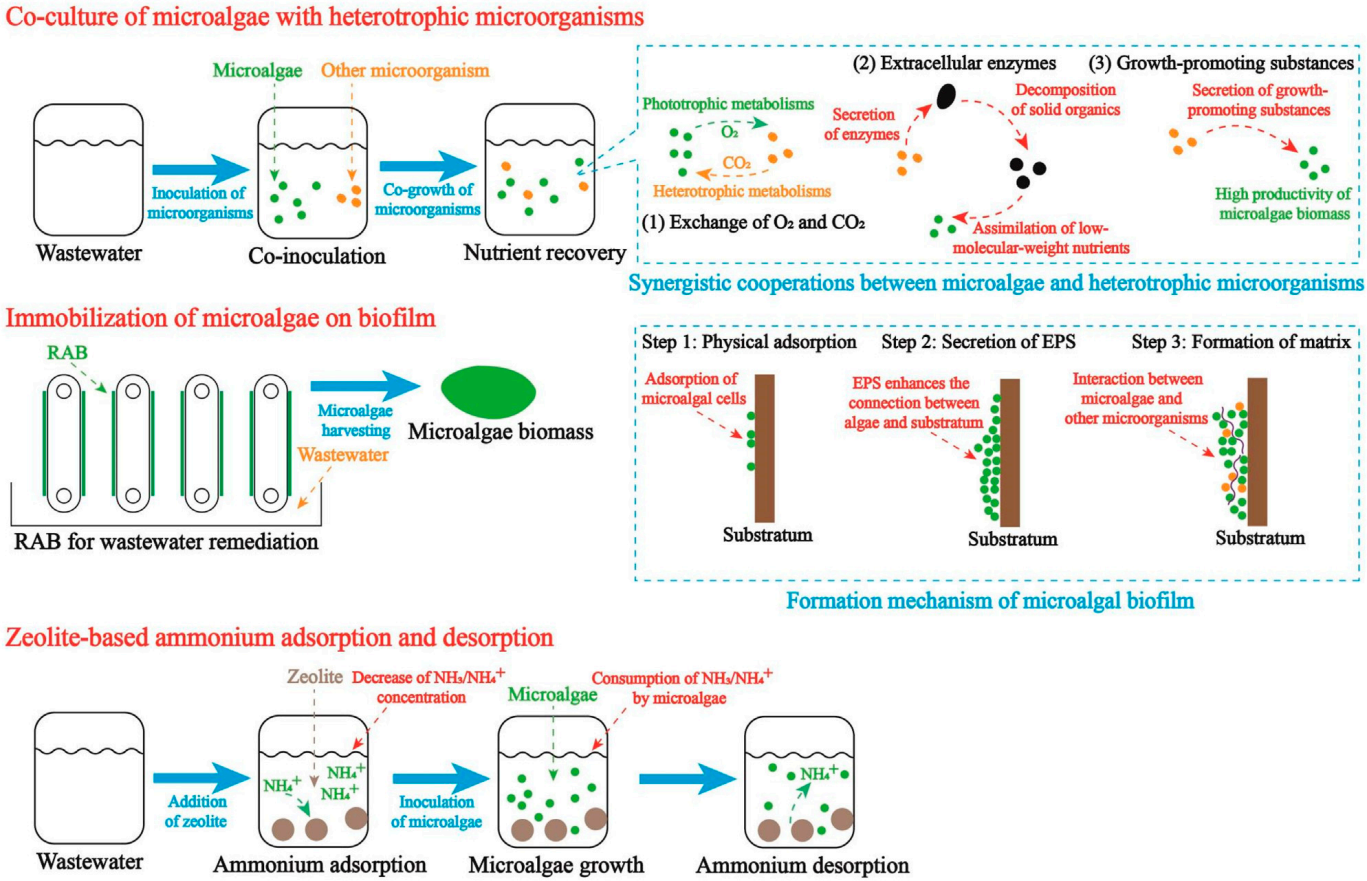
Technological innovations for microalgae-based FPE valorization.

Nutrient removal efficiency is one of the important parameters, which are employed to assess the performance of wastewater remediation. It has been discovered that the co-culture of microalgae with heterotrophic microorganisms, particularly bacteria, yeast, and fungi, could promote the organics decomposition and nutrients removal in wastewater remediation (Liu et al., 2017; Lu et al., 2023b). As shown in Figure 2, the synergistic cooperations between microalgae and heterotrophic microorganisms mainly include the interspecies exchange of O_2_ and CO_2_, organics decomposition accelerated by extracellular enzymes, and secretion of growth promoting substances (Lee C. et al., 2019). Firstly, the interspecies exchange of O_2_ and CO_2_ creates a “comfortable” environment for the growth of microalgae and heterotrophic microorganisms. In the co-culture system, CO_2_ produced by heterotrophic microorganisms could be captured by microalgae for photosynthesis while O_2_ released by microalgae is essential to the metabolisms of heterotrophic microorganisms (Lu et al., 2023a; Lu et al., 2023b). In contrast, without the interspecies exchange of O_2_ and CO_2_ between microalgae and heterotrophic microorganisms, algal cells would assimilate the atmospheric CO_2_, of which the low concentration and dissolution rate may limit the photosynthesis and biomass production. Secondly, some extracellular enzymes, such as protease, amylase, and lipase, secreted by bacteria and fungi can accelerate the organics decomposition, converting high-molecular-weight nutrients to low-molecular-weight nutrients. Accordingly, in the wastewater, an increasing amount of digestible nutrients will be available to microalgae. It should be noted that with the organics decomposition, the presence of some sugars and acids (glucose, fulvic acid, *etc.*) in wastewater could increase the astaxanthin yield of microalgae (Zhao et al., 2019a; Zhang et al., 2019). Thirdly, some bacteria and fungi could secret growth-promoting substances, which can increase the biomass yield of microalgae. For example, an auxin-producing symbiotic bacterium, *Achromobacter* sp., with the function of enhancing *H. lacustris* growth at all growth stages by secreting indole-3-acetic acid was isolated (Lee C. et al., 2019). Therefore, it is a feasible strategy to enhance the astaxanthin production of microalgae grown in FPEs by co-culturing microalgae and bacteria/fungi with growth-promoting functions.

In the academic research, the positive effects of co-culture of microalgae with some heterotrophic microorganisms on nutrient removal and astaxanthin production have been documented (Jiang et al., 2018). The practical application of this innovative strategy, however, is challenged by a couple of potential problems. For example, bacteria with fast reproduction rate may compete with microalgae for nutrients in FPEs, resulting in low yield of algal biomass and astaxanthin. In some cases, the fast growth of bacteria or fungi may even lead to the failure of microalgae cultivation in FPEs. Additionally, in the industry, the traditional method of analyzing bacterial growth trend by quantifying colony-forming unit (CFU) is time-costing while it is expensive to conduct real-time monitoring of the bacterial growth trend in wastewater remediation. Therefore, in the coming future, by realizing the real-time monitoring of bacterial growth at low cost, co-cultivation of microalgae (*H. lacustris* and *C. zofingiensis*) in FPEs for astaxanthin production might become more and more applicable in the industry.

### 4.2 Immobilization of microalgae on biofilm

In the past, most studies treated wastewater by directly inoculating the suspended microalgae cells while rarely focused on the utilization of immobilized microalgae cells for wastewater treatment. However, in the practical application, wastewater treatment by suspended microalgae is challenged by the high cost and high energy consumption of biomass harvesting. It was reported that energy consumption of centrifugation and filtration for microalgae harvesting could reach 1.43 and 1.22 kWh m^-3^, respectively (Najjar and Abu-Shamleh, 2020). Accordingly, the cost of microalgae harvesting is high, accounting for about 30% of the total cost of microalgae cultivation (Hu et al., 2021).

To use the algal astaxanthin as an ingredient of animal feed, the total cost of microalgae should be maintained at a low level and the safety of the harvested biomass must be improved. Recently, revolving algal biofilm (RAB), which can realize the immobilization of algal cells, is developed as an innovative method for microalgae cultivation and harvesting (Hu et al., 2021). In the RAB, microalgae are attached and grow on the surface of substratum (cotton duct, cotton rag, cotton denim, *etc.*) and the biomass harvesting can be conducted by using scrappers (Gross et al., 2013; Gross et al., 2015; Hu et al., 2021). Compared to centrifugation and filtration, RAB has much lower energy consumption for biomass harvesting and yields microalgal biomass with low cost. Therefore, RAB, which attracts attentions from researchers in both academia and industry, is being intensively studied and applied in the industry (Gross et al., 2013; Hu et al., 2021).

The formation of microalgal biofilm on substratum is mainly consisted of three steps (Figure 2). Firstly, microalgal cells are attached on the rough surface of substratum when the attractive van der Waals and acid-base interactions overcome the repulsive electrostatic interactions (Hu et al., 2021). In the practice, to enhance the adsorption of microalgal cells, substratum materials with rougher surface and more binding sites are preferred. Hence, many previous studies devoted a lot efforts to selecting the most appropriate substratum materials for microalgae adsorption (Shen et al., 2014; Ji et al., 2023). Secondly, microalgae attached on the surface of substratum could secret extracellular polymeric substances (EPS), which can act as “glue” in the formation of microalgae matrix (Hu et al., 2021). In this process, the presence of EPS could promote the construction of polymer network and improve the physical stability of microbial matrix on the surface substratum. Thirdly, with the gradual colonization of bacteria and filamentous fungi, the interaction between microalgae and substratum will be enhanced and the biofilm thickening process will be accelerated.

Up to now, RAB has been adopted to cultivate microalgae for nutrients recovery in a variety of waste streams, synthetic wastewater and artificial media. For example, in the trough-based RAB, maximum surface biomass productivity and footprint biomass productivity of *Chlorella vulgaris* reached 5.5 g m^-2^ day^-1^ and 46.8 g m^-2^ day^-1^, respectively (Gross et al., 2015). In terms of footprint biomass productivity, RAB, which can make full use of vertical space, has much better performance than traditional cultivation systems (Yu et al., 2024). In addition, since microalgae attached on substratum could be harvested easily by using scrappers and the harvested biomass has low moisture content, the cost of biomass harvesting and thickening can be reduced remarkably. Therefore, immobilization of microalgae by RAB to improve the footprint biomass productivity and simplify the biomass harvesting process is regarded as a promising strategy to upgrade microalgae-related industry and promote the commercialization of algal bioproducts.

Previous studies confirmed the feasibility of growing cylindrical or spherical microalgae, such as *Tetradesmus obliquus* (former name *Scenedesmus obliquus*) and *C. vulgaris*, on substratum for the construction of algal biofilm (Gross et al., 2015; Nguyen et al., 2023). The cells of *H. lacustris* and *C. zofingiensis* are also spherical, having similar size and structure with the cells of *T. obliquus* and *C. vulgaris*. In addition, both *H. lacustris* and *C. zofingiensis* have the ability to synthesize and secret EPS, which is one of the key compounds during the formation of algal biofilm (Delattre et al., 2016; Gorgich et al., 2021). Therefore, theoretically, *H. lacustris* and *C. zofingiensis* could be cultivated on RAB for astaxanthin production. Since RAB and relevant technologies are recently developed, up to now, few studies have comprehensively evaluated the astaxanthin production in RAB. In the future, experimental studies should be conducted to evaluate the specific effects of microalgae immobilization on astaxanthin synthesis and accumulation in RAB, providing practical guidance to the astaxanthin production.

Although RAB has obvious advantages over conventional microalgae cultivation modes, its drawbacks, such as washout of microalgal cells and poor penetration of light, should not be neglected. Due to the water flow and mechanical vibration, a portion of microalgal cells attached on the surface of biofilm substratum may fall into the wastewater (Zhao et al., 2018). With the discharge of FPE after the microalgae-based treatment, the algal cells suspended in FPE enter the natural waters. Hence, the occurrence of microalgae washout during the continuous operation of RAB not only lowers the final yield of biomass, but also causes potential biological invasion (Hu et al., 2021). In addition, thick layer of microalgae on biofilm substratum limits the penetration of light and the microalgal cells at the inner layer would not receive sufficient light. Accordingly, the insufficient illumination may negatively impact the synthesis of astaxanthin in the microalgal cells at the inner layer. Up to now, few studies have developed applicable methods to address the aforementioned drawbacks of RAB. In the view of the present authors, in the coming future, methods to trigger the secretion of EPS of microalgae could be employed to enhance the stickiness of the algal biofilm and prevent the washout of microalgal cells. Besides, artificial intelligence (AI) technology may be adopted to control the thickness of algal biofilm and promote the penetration of light.

### 4.3 Zeolite-based ammonium adsorption and desorption

It has been widely documented that high C/N ratio in culture media or waste stream is of importance to the induction of astaxanthin synthesis in microalgal cells (Río et al., 2005; Lu and Lu, 2022). Accordingly, FPEs with high concentration of TN, particularly TAN, were rarely utilized for microalgae-based astaxanthin production. In addition, during the algae growth process, with the exhaustion of ammonia in FPEs, extremely low concentration of nitrogen in the later stage may be unfavorable to algal cells. Therefore, to widely apply FPEs as an alternative substrate for microalgae cultivation and astaxanthin production, the initial concentration of TN and the ratio of C/N in FPEs should be regulated properly.

In the past, to attenuate ammonia toxicity or adjust the ratio of C/N in wastewater, some methods, such as dilution by freshwater and ammonia stripping, have been developed (Li J. et al., 2019; Lu et al., 2023b). However, in the practical application, these traditional methods are hindered by a couple of problems. Firstly, concentrations of TN and TAN in waste stream or culture media can be effectively reduced via dilution. However, dilution by freshwater not only reduce the concentrations of TAN and TN, but also lower the concentration of TOC in waste stream or culture media. As a result, the ratio of C/N in FPEs may not be improved obviously and the astaxanthin synthesis in microalgae will not be positively impacted. Besides, dilution, which consumes a large volume of freshwater, can not be regarded as an eco-friendly and sustainable method for the pretreatment of FPEs (Lu et al., 2023a). Secondly, as a process releasing dissolved ammonia into atmosphere, ammonia stripping can effectively reduce the concentrations of TN and TAN in FPEs while have no obvious effect on the concentration of TOC. This method, hence, can be employed to increase the ratio of C/N in FPEs, creating a suitable environment for microalgae-based astaxanthin production. However, ammonia stripping is accompanied with intensive emission of ammonia, not only reducing the utilization efficiency of TN in FPE, but also cause air pollution (Lu et al., 2023b). Besides, due to the low concentrations of TN and TAN in FPE after ammonia stripping, microalgae may suffer from the nitrogen deficiency in the later stage of exponential phase. Because of the disadvantages mentioned above, technological innovation is needed to realize the microalgae-based astaxanthin production in FPE.

Zeolite is a porous material which can effectively adsorb the ionized ammonium (NH_4_^+^) when the concentration of ammonium in water is high while slowly release NH_4_^+^ when ammonium is exhausted. Firstly, at the initial stage, with the addition of zeolite in FPE, NH_4_^+^ is adsorbed and the concentrations of TAN and TN would be dramatically reduced. As reported by previous study, when zeolite was added in wastewater (960–1,080 mg L^-1^ TAN) at a density of 125 g L^-1^, after 15-h, 71.27% of TAN was transferred into zeolite, resulting in a low concentration of TAN (285 mg L^-1^). Accordingly, the ratio of TOC to TAN in wastewater was remarkably increased from 4.83 to 16.77 (Lu et al., 2023a). Hence, with the addition of zeolite, ammonia toxicity could be attenuated and FPE with high C/N ratio is more favorable to the astaxanthin synthesis in algal cells. Secondly, at the later stage, with the gradual consumption of TAN and further increase of C/N ratio, NH_4_^+^ is released from zeolite to maintain the C/N ratio in FPE stable. In our previous study, due to the slow-release of NH_4_^+^ by zeolite, TAN concentration in media was maintained stably in a range of 40–50 mg L^-1^ at the later stage (Day 8 to Day 18) (Lu et al., 2019). Accordingly, the limiting effect of ammonia exhaustion on algal cells can be alleviated, resulting high yields of algal biomass and astaxanthin. Based on the mechanisms and experimental data mentioned above, zeolite-based ammonium adsorption and desorption is regarded as a promising strategy to promote the practical application of FPE for microalgae cultivation and astaxanthin production.

Up to now, dynamics of zeolite-based ammonium adsorption and desorption have been intensively studied and this novel strategy has been successfully implemented to regulate the C/N ratio for *Arthrospira platensis*, *Amphidinium carterae*, and *C. vulgaris* cultivation in waste streams (Markou et al., 2014; López-Rosales et al., 2022; Lu et al., 2023a). It was reported that due to the improvement of C/N ratio in wastewater, oil yield and oil content of *C. vulgaris* were improved to 1.24 g L^-1^ and 34.6%, respectively (Lu et al., 2023a). These results fully confirmed the practical feasibility of enhancing the biosynthesis of certain algal compounds by zeolite-based ammonium adsorption and C/N adjustment.

Due to the close relation between C/N ratio and astaxanthin synthesis, zeolite-based ammonium adsorption and desorption can play a key role in the microalgae-based FPE remediation for astaxanthin production. Theoretical procedures are shown in Figure 2. Firstly, based on the optimal C/N ratio for astaxanthin in microalgae, the adding amount and adsorption time of zeolite in FPE should be identified. At this step, physical structure of zeolite, pH of wastewater, temperature, and concentrations of ions in wastewater can impact the dynamic equilibrium and adsorption rate of ammonium. Secondly, by the end of ammonium adsorption, microalgae are inoculated in FPE for nutrient assimilation and astaxanthin production. With the gradual consumption of TAN by microalgae, adsorbed ammonium is released from zeolite into FPE, continuously maintaining the TAN concentration and C/N ratio in wastewater. Thirdly, by the end of FPE remediation, algal biomass enriched with astaxanthin is harvested for downstream application.

One of the major drawbacks of this technology is that the management of zeolite in wastewater treatment is a labor-intensive process. Firstly, according to the data in previous study, total weight of zeolite used for ammonia adsorption is high (Zhao et al., 2018). For example, if the adding amount of zeolite in wastewater is set as 125 g L^-1^, to treat 1,000 m^3^ wastewater simultaneously, 125 tons zeolite will be used. Undeniably, it is a labor-intensive process to transport and manage such a huge amount of zeolite. Secondly, to reduce the total cost of zeolite-based wastewater treatment, the used zeolite should be collected and then washed for reuse (Lu et al., 2019). Such a process can be time-consuming and labor-intensive, limiting the wide application of zeolite in FPE remediation. In the view of the present authors, in the further research, efforts could be devoted to find out the zeolite with lower density and higher adsorption capacity for FPE treatment. Additionally, synthetic materials with better performance in ammonia adsorption and desorption could be developed to replace zeolite for FPE treatment.

## 5 Downstream application of microalgal astaxanthin in agriculture

### 5.1 Selection of the target market

Based on the discussion above, astaxanthin obtained from the microalgae cultivation in FPE has the advantages of low cost and no toxicity. To truly promote the long-term development of microalgae cultivation in FPE for astaxanthin production, it is of necessity to select the appropriate target market.

At present, the industries, which have high demand on astaxanthin, mainly include cosmetics, food, and animal feed (Lu et al., 2021; Lu and Lu, 2022). Firstly, astaxanthin with antioxidative properties could be added in cosmetics to attenuate the cellular senescence. Low cost of the raw material, however, is rarely a crucial concern of the cosmetics manufacturers due to the high profitability of cosmetics products. Thus, low-cost microalgal astaxanthin obtained from FPE might not be attractive to the manufacturers. Secondly, although microalgal astaxanthin obtained from FPE treatment has no toxicity, it may not be widely accepted by consumers. In the traditional mindset, the sources of food ingredients should never be the FPE. Thirdly, the increase of global population has stimulated the fast development of livestock and poultry farming and aquaculture. Accordingly, market demand on animal feed is increasing continuously, providing numerous opportunities to the commercialization of microalgal astaxanthin. Besides, microalgal astaxanthin could enhance the immune response of animals and prevent the overuse of antibiotics or medicines (Xie et al., 2018; Xie et al., 2020). The addition of microalgal astaxanthin in animal feed, hence, would be supported by the government, industry, and consumers. Last but not the least, due to the low cost, microalgal astaxanthin obtained from FPE is affordable to the manufacturers of animal feeds.

In recent years, owing to the promising application potential of microalgal astaxanthin in animal feed, the industry of animal feed production has become one of the major target markets. In addition, feed recipe research and animal experiment have been intensively conducted to promote the commercialization of microalgal astaxanthin. Hence, in the market, exploitation of algal astaxanthin as an ingredient of animal feed is emerging into the limelight.

### 5.2 Supplementation of microalgal astaxanthin in animal feed

Up to now, microalgal astaxanthin has been successfully applied in livestock feeding, fish and shrimp feeding, and poultry feeding. Dietary supplementation of astaxanthin in animals feeding could positive impact the meat quality, immune response, and growth rate (Table 4). Accordingly, the overuses of antibiotics and medicines in animal farming industry could be prevented. This is of importance to the development of eco-friendly agriculture and production of high-quality meat products.

**TABLE 4:** Application of microalgal astaxanthin for animals feeding.

Application field	Animal	Algal species	Form of astaxanthin	Concentration of astaxanthin	Specific effects	References
Livestock farming	Weaned pig	*H. lacustris*	Astaxanthin extract	0.025 g kg^-1^ diet	1) Supplementation of astaxanthin in pig diet improved the shelf life of pork fat; 2) The growth performance of pigs fed the astaxanthin did not differ from pigs fed a control diet.	Szczepanik et al. (2022)
	Finishing pig	*NA ^a^*	Astaxanthin	1.5 and 3.0 ppm of astaxanthin in diet	1) Dietary supplementation of astaxanthin in diet reduced the cholesterol content in the meat of finishing pig; 2) Dietary supplementation of astaxanthin in diet improved the carcass traits and meat quality of finishing pig	Yang et al. (2006)
	Heifer	*NA*	*NA*	0.25 mg astaxanthin per kg BW per day per animal	1) Dietary supplementation of astaxanthin increased the body weight gain and feed intake; 2) Feed conversion ratio was reduced by the supplementation of astaxanthin in diet.	Kumar et al. (2019)
Aquaculture	Rainbow trout (*Oncorhynchus mykiss*)	*C. vulgaris*	Algal biomass	Diet was supplemented with 4% algal biomass containing 0.2% carotenoids (30% astaxanthin)	1) Dietary supplementation of algal biomass did not significantly change the total feed intake and weight gain; 2) Accumulation of carotenoids (11.9 mg kg^-1^ dry muscle) in the muscle of fish was observed; 3) Compared with synthetic pigments, algal biomass is a slightly less efficient muscle coloring ingredient for farmed trout	Gouveia et al. (1997)
	Chinese mitten crab (*Eriocheir sinensis*)	*H. lacustris*	Defatted algal meal	Diet containing 1% defatted algal meal (64.80 mg astaxanthin/kg dry diet)	1) Malonaldehyde contents in the serum and the hepatopancreas of male crab were reduced to 3.92 nmol mL^-1^ and 1.64 nmol mg^-1^ protein; 2) The contents of astaxanthin and total carotenoids in the carapace of crab were improved; 3) Astaxanthin-rich algal meal significantly reduced the cost of feed formulation, improved the coloration, enhanced antioxidant and immune capacity of adult crab	Ma et al. (2019)
	Rotifer (*Brachionus plicatilis*)	*H. lacustris*	Defatted algal meal	Addition of defatted algal meal (containing 0.5% *w*/*w* astaxanthin) at a content of 125 mg L^-1^	1) The density of rotifer fed astaxanthin-rich algal meal was higher than that of control group; 2) When the addition of algal meal was 125 mg L^-1^, rotifer egg density was the highest; 3) The contents of astaxanthin and carotenoid in rotifer were improved to 0.60 and 0.76 mg g^-1^ (wet weight)	Li and Liu (2019)
	Rainbow trout (*Oncorhynchus mykiss*)	*H. lacustris*	Commercial algal biomass	Addition of algal biomass in diet at a content of 2.80, 5.60, and 11.20 g kg^-1^	Dietary supplementation of astaxanthin-rich algal biomass improved the fillet quality of rainbow trout via attenuating the oxidative stress and functions of antioxidant-relevant enzymes, thus protecting fish from arsenic toxicity	Milan et al. (2021)
	Post-larval white shrimp (*Litopenaeus vannamei*)	*H. lacustris*	Algal biomass	Algal biomass (astaxanthin content: 30 g kg^-1^ dry weight) was added in the diet at a content of 3.3 g kg^-1^	1) Astaxanthin-rich algal biomass increased the survival rate (72.08%) of post-larval white shrimp; 2) After the acute salinity stress, shrimp fed with algal astaxanthin had lower malonaldehyde content; 3) Dietary supplementation of astaxanthin-rich algal biomass increased the anti-oxidative ability and immune capacity of shrimp	Xie et al. (2018)
	Golden pompano (Trachinotus ovatus)	*H. lacustris*	Algal biomass	Content of algal biomass in diet was 0.3%	1) Compared to the fish in control group, the fish fed with astaxanthin-rich algal biomass had higher weight gain and specific growth rate; 2) Dietary supplementation of astaxanthin-rich algal biomass increased the protein content in the whole-body compositions; 3) Astaxanthin-rich algal biomass alleviated the inflammatory response of fish under acute hypoxia stress by activating Nrf2-ARE pathway to antagonize the NF-κB pathway	Xie et al. (2020)
Poultry farming	Broiler chicken	*H. lacustris*	Algal meal	Addition of 350, 1800, and 8,950 mg algal meal/kg feed (7, 36, and 179 mg astaxanthin/kg feed)	1) The contents of astaxanthin and total carotenoids in liver, kidney, intestine, and breast muscle increased; 2) Astaxanthin-rich algal meal supplement did not influence growth performance, feed intake or feed conversion ratio	Waldenstedt et al. (2003)
	Laying hens	*H. lacustris*	Astaxanthin extract	20, 40, 80, and 160 mg astaxanthin/kg diet	1) The increase of dietary astaxanthin did not significantly impact the egg weight, laying rate, feed consumption, eggshell strength, feed efficiency, or Haugh unit; 2) The scavenging abilities of hydroxyl radicals and superoxide anions were linearly increased with the increase of astaxanthin level; 3) Yolk color darkened linearly with the increase of astaxanthin level	Heng et al. (2021)
	Broiler chicken	*H. lacustris*	Astaxanthin powder	10, 20, 30, and 40 mg astaxanthin powder/kg feed	Dietary supplementation of astaxanthin to the broiler meat diet resulted in an improvement in the immune characteristics of broilers at normal and elevated environmental temperatures	Awadh and Zangana (2021)
	Laying hens	*H. lacustris*	Astaxanthin extract	20, 40, 80, and 160 mg astaxanthin/kg diet	Dietary supplementation of astaxanthin extracted from microalgae delayed the decrease in yolk index and yolk color during the storage at 4°C and 25 °C	Heng et al. (2020)
	Layers	*NA*	Astaxanthin	0.7, 0.9, 1.1 and 1.3 ppm of astaxanthin in diet	1) Dietary supplementation of astaxanthin in poultry diet had no significant effect on layer production performance; 2) Yolk colorness was linearly increased with the increase of astaxanthin content in poultry diet.	Yang et al. (2006)
	Layer hens	*H. lacustris*	Full fatted microalgae	Basal diet was supplemented with 0.08% and 0.32% of algae to provide 20 and 80 mg of astaxanthin/kg of diets	1) Dietary supplementation of astaxanthin resulted in the enrichment of astaxanthin and total carotenoids in the plasma and egg yolk of hens; 2) Egg yolk color was changed by the addition of microalgal astaxanthin in diet; 3) The increase of astaxanthin content in diet reduced the malondialdehyde content in liver and egg yolk	Magnuson et al. (2018)

^a^ *NA* means not available.

#### 5.2.1 Enrichment of astaxanthin

Dietary supplementation of microalgal astaxanthin could directly enrich astaxanthin in animal meat, improving the anti-oxidative properties and carcass traits of animal products. When *Chlorella* was added in fish diet for rainbow trout (*Oncorhynchus mykiss*) culture, accumulation of carotenoids (11.9 mg kg^-1^ dry muscle) was detected in the muscle of fish after 9 weeks (Gouveia et al., 1997). Similar phenomena were discovered in rotifer (*Brachionus plicatilis*) fed defatted *H. lacustris* meal and yolk colorness of layer hens fed algal astaxanthin (Magnuson et al., 2018; Li and Liu, 2019; Heng et al., 2021). With the enrichment of astaxanthin, anti-oxidative properties and carcass traits of animal products were improved. Firstly, since algal astaxanthin is beneficial to the health of humans, with the daily intake of astaxanthin-rich meat or egg, the health of consumers will be positively impacted. Secondly, pigmentation and brightness of in meat can be improved by the enrichment of astaxanthin, positively impacting the carcass traits and attractiveness of animal products (Yang et al., 2006). Therefore, dietary supplementation of microalgal astaxanthin can improve the qualities and market values of some animal products.

Deterioration is one of the main factors resulting in the short shelf life of meat and egg products. Besides, deterioration may cause the formation of oxidation compounds, such as aldehydes and ketones, with unfavorable flavors and unhealthy effects. As a consequence, the quality of meat and egg would be negatively impacted and the health of consumers would be threatened. At present, cold-chain logistics is adopted to reduce the deterioration rate and prolong the shelf life of meat and egg products but its cost is extremely high. In previous studies, it was discovered that the enrichment of algal astaxanthin in meat and egg could effectively attenuate deterioration and extend the shelf life of products (Heng et al., 2020; Szczepanik et al., 2022). For example, with the addition of algal astaxanthin in chicken diet, the decrease in yolk index and yolk color was delayed during the storage at 4°C and 25 °C (Heng et al., 2020). Besides, the improvement of shelf life of pork fat by dietary supplementation of algal astaxanthin in pig diet was reported (Szczepanik et al., 2022). The main mechanism for this phenomenon is that astaxanthin with super anti-oxidative capacity would react with oxidative substances in priority, thus protecting the meat and egg products from deterioration (Yuan et al., 2011).

#### 5.2.2 Reduction of oxidative stress

Reactive oxygen species (ROS) are accumulated in cells and oxidative stress is improved when livestock, fish, shrimp and poultry are exposed to harsh environment. As reported by previous study, malonaldehyde (MDA) content of the whole shrimp body increased from 0.12 to 0.23 nmol mg^-1^ prot when post-larval white shrimps were exposed to salty stress (Xie et al., 2018). To improve the health status and survival of animals in the harsh environment, ROS should be scavanged properly.

Astaxanthin is a superantioxidant with the capacity of scavenging ROS in animals suffered from unfavorable environment. For example, with the dietary supplementation of defatted *H. lacustris* meal, MDA contents in the serum and hepatopancreas of adult Chinese mitten crab *Eriocheir sinensis* were reduced to 3.92 nmol mL^-1^ and 1.64 nmol mg^-1^ protein, respectively (Ma et al., 2019). In addition, after the acute salinity stress, compared with the control group (0.23 nmol MDA mg^-1^ protein), white shrimp (*Litopenaeus vannamei*) fed *H. lacustris* had much lower MDA content (0.16 nmol mg^-1^ protein) (Xie et al., 2018). Particularly, via scavenging ROS and reducing oxidative stress, survival of white shrimp was remarkably improved (Xie et al., 2018). Similarly, positive effects of dietary supplementation of algal astaxanthin on immune characteristics of broilers were reported (Awadh and Zangana, 2021). For example, the increase of algal astaxanthin content in chicken diet even caused the drop of MDA content in egg yolk (Magnuson et al., 2018).

It should be noted that the adding amount of algal astaxanthin in diet should be optimized according to the experimental results. In the culture of post-larval *L. vannamei*, before the acute salinity stress, the increase of *H. lacustris* content in diet from 3.3 to 13.3 g kg^-1^ did not cause the significant drop of MDA content in whole shrimp body (Xie et al., 2018). Hence, in terms of the reduction of oxidative stress, excessive addition of algal astaxanthin in animal diet may cause the waste of astaxanthin ingredient.

#### 5.2.3 Promotion of animal growth

In addition to enhancing pigmentation and reducing oxidative stress, in some studies, dietary supplementation of astaxanthin-rich microalgae could effectively promote animal growth. It was discovered that feed intake and body weight gain of heifers increased with the addition of astaxanthin in diet and feed conversion ratio (FCR) was reduced accordingly (Kumar et al., 2019). Positive effects of dietary supplementation of algal astaxanthin on the weight gain and specific growth rate of golden pompano (*Trachinotus ovatus*) were also reported (Xie et al., 2020). According to the aforementioned results, microalgal astaxanthin can be employed as a growth-inducing agent in animal feed.

Nevertheless, it is noteworthy that some studies did not find the positive relation between dietary supplementation of algal astaxanthin and animal growth. In the animal experiment, when the addition of algal astaxanthin in chicken diet was 0 and 160 mg kg^-1^, feed consumption reached 136.30 and 137.40 g day^-1^, respectively (Yang et al., 2006). This result demonstrates that microalgal astaxanthin could not significantly improve the feed consumption of laying hens. In the culture of weaned pig, supplementation of algal astaxanthin at a concentration of 0.025 g kg^-1^ in diet did not significantly improve the average daily weight gains or reduce the FCR (Szczepanik et al., 2022). In the view of the present authors, in some cases, negative effect of astaxanthin-rich microalgae on animal growth is partially attributed to the poor digestability of algal biomass. Cellulose, which is one of the major components in the cell wall of microalgae, could not be efficiently digested by some carnivorous fish, thus resulting in the low weight gain and survival efficiency of *Micropterus salmoides* fed the diet with high content of microalgae (Deng et al., 2021).

Up to now, the mechanisms for the positive effects of algal astaxanthin on animal growth have not been fully revealed. Due to the contradictory opinions in previous studies, in the practice, the specific effects of algal astaxanthin on animals should be assessed on a case by case basis.

Based on the discussion above, in some cases, the addition of algal astaxanthin in diet could promote the enrichment of astaxanthin in meat and egg products, enhance the immune response, and improve the growth rate of animals. Compared to antibiotics and artificial hormone, algal astaxanthin, a type of natural pigment with anti-oxidative and growth-enhancing properties, has much less side effects.

## 6 Problems and prospects

The practical application of FPE for microalgae cultivation and astaxanthin production and the use of astaxanthin-rich microalgae as a feedstock for animal feed production are challenged by a couple of potential problems, including the potential safety risks of FPE in logistics, lack the pilot-scale experiments, and the poor degistibility and palatability of algal biomass. In the view of the present authors, to improve the contribution of algal astaxanthin to eco-friendly agriculture, it is of necessity to reduce the total cost of algal astaxanthin, make policy to support the use of FPE as medium alternative, and promote the technological innovations.

### 6.1 Potential problems in the practical application

#### 6.1.1 Potential safety risks of FPE during logistics

To prevent the accumulation of unhealthy or toxic compounds in food chain, FPE, which is employed for microalgae cultivation, should not contain unhealthy or toxic compounds. Although most FPEs are regarded as nutrient-rich waste stream containing no toxic compounds, some FPEs might be contaminated by toxic bacteria. For example, in the lab research, researchers obtain low volume of FPE from factory directly and store FPE in refrigerator before the experiment. By contrast, in the industrial application, various bacteria in FPE can have much higher growth rates during the long-distance transportation. With the growth of toxic bacteria or the secretion of toxic compounds by bacteria in FPE, microalgae cultivated in FPE will be contaminated, challenging the safety of microalgae-based diet. According to the previous studies, toxic or unhealthy bacteria discovered in the algal-bacteria consortia include and so on. To our knowledge, due to the potential safety risks, few countries or regions have made policies or regulations to support the wide use of microalgae cultivated in wastewater as the feedstock for animal feed production.

Although cold-chain logistics can effectively prevent the fast growth of bacteria in FPE during the transportation and storage, it can remarkably increase the total cost of microalgae cultivation. Additionally, due to the large volume of FPE in the practice, in most cases, it is too hard to employ the cold-chain logistics to attenuate the deterioration of FPE in the transportation and storage. Therefore, in terms of the safety risks of FPE, there is a huge gap between lab research and industrial application. Based on the present technologies, with the fast growth of bacteria in FPE during transportation and storage, FPE may not be suitable to be used for microalgae cultivation.

#### 6.1.2 Lack of pilot-scale experiments

Technological innovations, such as algal-bacterial co-culture, immobilization of microalgae, and zeolite-based ammonium adsorption, mentioned above have been intensively studied in the lab, however, they are rarely applied in the industry. For example, mechanisms associated with the microalgae immobilization on biofilm for wastewater remediation have been revealed in the lab research, but only a few studied conducted the pilot-scale experiments to evaluate the practical feasibility of RAB for algal astaxanthin production. Also, the concept of zeolite-based ammonium adsorption and desorption was mainly studied in the lab research while pilot-scale experiments were rarely conducted to use this innovative method for astaxanthin-rich microalgae cultivation (Lu et al., 2019; Lu et al., 2023a). Due to the lack of pilot-scale experiments, it is too hard to find out the weaknesses of these innovative methods developed in lab research and making them to be more applicable in the industry.

#### 6.1.3 Poor digestibility and palatability of microalgal biomass

The cell wall of microalgae is consisted of cellulose and hemicellulose, which can not be digested efficiently by some carnivorous fish. In the aquaculture practice, poor digestibility of microalgal biomass results in the high FCR, low growth rate, and high cost of aquaculture production. Under this situation, even if microalgal biomass is enriched with a variety of high-value compounds, including natural astaxanthin, the nutrients contained in microalgae may not be fully assimilated by animals. In addition, fishy smell, which can increase the feed attraction and improve the feed intake, is an important factor impacting the quality of fish diet. In terms of the palatability, microalgae without fishy smell can not be regarded as a promising ingredient in fish diet. According to the previous studies, higher inclusion ratio of microalgal biomass in fish diet is always accompanied with lower feed intake (Deng et al., 2021). Due to the poor digestibility and palatability of microalgae, the application of microalgal biomass as a major ingredient in fish diet is hindered.

The straightforward strategies to improve the digestability of astaxanthin-rich microalgae include breaking the structure of cell wall and extracting astaxanthin from algal biomass. For example, cellulases can be employed to accelerate the decomposition of cell wall of microalgae and promote the release of astaxanthin contained in algal cells. Besides, since astaxanthin is a fat-soluble pigment, oil extraction method can be adopted to separate astaxanthin from algal biomass. Then, the astaxanthin-containing oil is added in diet to provide algal astaxanthin to animals (Namekawa et al., 2010). However, the aforementioned methods have high requirements on the technologies and increase the total cost of astaxanthin and astaxanthin-containing feed.

### 6.2 Prospects of the application of algal astaxanthin

#### 6.2.1 Reduction of the total cost of algal astaxanthin

High price in the market is one of the major factors that limit the wide application of algal astaxanthin in agriculture. In many developing countries, since agricultural products are sold at low price, farmers or agricultural companies are not willing to use algal astaxanthin in animal diet to develop the eco-friendly agriculture even if they realize the ecological and environmental benefits of algal astaxanthin. In the coming future, to improve the market acceptance of algal astaxanthin, measures must be taken to further reduce the total cost of algal astaxanthin.

In the view of the present authors, in addition to the replacement of artificial medium by FPE, other methods, such as the use of solar energy for illumination and the increase of astaxanthin content in microalgae by genetic modification, could be applied to further lower the total cost of algal astaxanthin. It is expected that with the drop of production cost of algal astaxanthin, the acceptance of astaxanthin-based products in the market will be remarkably improved.

#### 6.2.2 Policy support from the governments

In most developing countries, due to the lack of policies associated with the volarization of waste streams, most farms and food processing industries are not willing to recycle FPE for microalgae cultivation. The main reasons for this phenomenon include the high investment of microalgae cultivation systems, uncertainty in the market of algal bio-products, and lack of advanced technologies for astaxanthin exploitation. In addition, compared with antibiotics, medicines, and artificial hormones, which have been widely used in animal farming for a long time, algal astaxanthin is much less popular in the market. Accordingly, the concept of employing FPE for astaxanthin-rich microalgae cultivation is not attractive to the managers of food processing factories and farms.

In the foreseeable future, to promote the fast development of FPE valorization, astaxanthin-based products, and eco-friendly agriculture, governments should make appropriate policies. For example, the abuses of antibiotics, medicines, and artificial hormones in animals farmings should be forbidden by laws. Only in this way could the managers of farms start to focus on the use of eco-friendly bio-products for animals farming. In addition, policies should be made to require food processing industry to monitor the quality of FPE during transportation and storage, ensuring that microalgae cultivated in FPE would not be contaminated by toxic compounds or unfavorable bacteria. In this way, the quality of astaxanthin produced by FPE-based microalgae cultivation can be improved and the astaxanthin-based products can be more attractive to the consumers in the downstream industry.

#### 6.2.3 Further improvement of the innovative technologies

Specific methods to apply the innovative technologies for microalgae-based astaxanthin production in FPE should be developed based on the pilot-scale experiments. Firstly, research interests should be shifted from the exploration of mechanisms to pilot-scale system design and operation gradually. Secondly, factors, such as complexity of technologies, total cost, availability of raw materials, and so on, which are important to the practical production and application of algal astaxanthin should be taken into consideration by researchers. For example, in previous studies, zeolite-based ammonium adsorption and desorption were conducted in flasks for microalgae cultivation in waste streams while the effectiveness of this innovative technology in pilot-scale system was remained unclear (Lu et al., 2019; Lu et al., 2023a). Besides, techno-economic analysis has not been conducted to comprehensively assess the cost and profitability of employing zeolite for microalgae cultivation in ammonium-rich waste streams. In other words, current research results could not fully present the advantages of zeolite-based ammonium adsorption and desorption for microalgae cultivation and astaxanthin production in FPE.

## 7 Conclusion

It is concluded that 1) *H. lacustris* and *C. zofingiensis*, which are enriched with astaxanthin, easily obtained, highly tolerant to harsh environment in wastewater, are regarded as promising microalgal strains for astaxanthin production in FPE; 2) FPEs, which contain essential nutrients for microalgae cultivation, can be regarded as a promising alternative to artificial medium; 3) based on the theoretical estimation, CO_2_ emission of microalgae-based FPE remediation is 79.89% lower than that of FPE treatment by chemical oxidation; 4) innovative technologies, including algal-bacterial co-culture, immobilization of microalgae, and zeolite-based ammonium adsorption, have been developed to promote the microalgae-based wastewater valorization; 5) Dietary supplementation of algal astaxanthin is beneficial to the weight gain, immune response, and pigmentation of animals, thus preventing the abuses of antibiotics, artificial hormones, and medicines in animals farming.

It is undeniable that in the practical application, the use of FPE for microalgae cultivation and astaxanthin is challenged by a couple of problems, such as the potential safety risks of FPE during logistics, lack of pilot-scale experiments, and poor digestibility and palatability of microalgal biomass. In the view of the present authors, efforts should be make to lower the total cost of algal astaxanthin, provide policy support, and further improve the innovative technologies, promoting the industrialization of FPE valorization by microalgae-based astaxanthin production.
